# ‘Why does it happen like this?’ Consulting with users and providers prior to an evaluation of services for children with life limiting conditions and their families

**DOI:** 10.1177/1367493513510630

**Published:** 2015-09

**Authors:** Anne Hunt, Erica Brown, Jane Coad, Sophie Staniszewska, Suzanne Hacking, Brigit Chesworth, Lizzie Chambers

**Affiliations:** University of Central Lancashire, UK; Coventry University, UK; Warwick Medical School, UK; University of Central Lancashire, UK; Together for Short Lives, UK

**Keywords:** Children’s participation, family, health services research, palliative care, quality of care

## Abstract

Children with life limiting conditions and their families have complex needs. Evaluations must consider their views and perspectives to ensure care is relevant, appropriate and acceptable. We consulted with children, young people, their parents and local professionals to gain a more informed picture of issues affecting them prior to preparing a bid to evaluate services in the area. Multiple methods included focus groups, face-to-face and telephone interviews and participatory activities. Recordings and products from activities were analysed for content to identify areas of relevance and concern. An overarching theme from parents was ‘Why does it happen like this?’ Services did not seem designed to meet their needs. Whilst children and young people expressed ideas related to quality of environment, services and social life, professionals focused on ways of meeting the families’ needs. The theme that linked families’ concerns with those of professionals was ‘assessing individual needs’. Two questions to be addressed by the evaluation are (1) to what extent are services designed to meet the needs of children and families and (2) to what extent are children, young people and their families consulted about what they need? Consultations with families and service providers encouraged us to continue their involvement as partners in the evaluation.

## Introduction

This article reports on consultations with children and young people with life limiting conditions, their parents and providers of services. The consultations were undertaken to inform a proposal for research (to become known as ‘The Big Study’) into the extent to which supportive and palliative care services meet the needs of life limited children and young people and their families in the West Midlands. Life limiting conditions are anticipated to result in the death of the child or young person in childhood or as a young adult. The upper age varies with demographic change and service policy, for example, life expectancy in boys and young men with Duchenne muscular dystrophy has increased significantly in the last 20 years (Fraser et al., 2012a). Because of limited appropriate adult services, children’s services may continue to provide supportive and palliative care and/or to develop new young people’s services to fill the gap. As long as the young people continued to receive care from children’s services (e.g. Community Children’s Nursing Services and Children’s Hospices), they and their families were eligible for inclusion in the consultation described here.

## Background

Although there remains considerable debate about the figures (Fraser et al., 2012b), it has been estimated that approximately 23,500 children and young people in the United Kingdom are living with a life limiting condition at any one time, and on average 16 per 10,000 children under 19 years of age require supportive and palliative care services (ACT, 2009). The Association for Children’s Palliative Care (ACT)^1^ has developed a series of integrated multi-agency care pathways to drive forward improvement in the delivery of supportive and palliative care services to infants, children and young people. Such pathways aim to ensure that children and young people are at the centre of service planning and development and that there is equity and quality of care irrespective of location or diagnosis.

Despite these aspirations, children and young people with palliative care needs, and their families, can still be marginalised (Bluebond-Langner, 1980, 1989; Carter and Coad, 2009; Frager and Collins, 2006). A recent review of children’s palliative care services (Craft and Killen, 2007) showed that there is still inequity of service provision across England and that there are challenges to the sustainability of services. The review highlighted that, whilst progress in medicine has led to many of these children living for longer, their lives are still often limited in quality. Care is often required from families over an extended period of time and hence appropriate domiciliary support is increasingly in demand. Consequently, caring for life limited children and their families has been refocused from hospital care to home care. There is, therefore, a need for better care in the community for an increased ability of community children’s nursing services to provide 24-hour support and for clear workload planning and integrated working (Craft and Killen, 2007; Department of Health, 2004, 2006; Glendinning et al., 2001; Hallett-Hughes et al., 2011; Lewis and Pontin, 2008). Despite attempts to address these issues, some children and families who require end-of-life care are still unable to access the *right care at the right time* (ACT, 2010).

### Involvement of children, young people and families in service development and evaluation

Over the last decade, patient and public involvement (PPI) in health and social care has grown nationally and internationally (Farrell, 1999; Staniszewska, 2009; World Health Organization, 2007). In the United Kingdom, there have been two linked but distinct areas of activity, PPI in health and social care services and PPI in health and social care research (Brett et al., 2012). The latter is essential for enhancing the quality, relevance and acceptability of research (Staley, 2009). The degree of PPI in research can vary from consultative forms of engagement to more collaborative forms and to user-controlled research in which service users take the lead in a study (INVOLVE, 2012; Morrow et al., 2010).

Whilst PPI in health and social care research has progressed markedly in the last decade, the evidence base underpinning it remains incomplete and often lacks coherence (Crawford et al., 2003; Staniszewska et al., 2008). Reporting is scarce and sparse, making it difficult to evaluate the quality of user involvement. Work is currently underway to address these issues (Staniszewska et al., 2011). There are now several published examples of consultation with children and young people with life limiting conditions around their perceptions of services and the choices they are offered and make. In relation to PPI in children’s palliative care research, although there are examples of consultations with parents (Edwards et al., 2011; Malcolm et al., 2009) and with health professionals (Steele et al., 2008) around priority setting for research, the involvement of the children and young people themselves is less frequently reported. The James Lind Alliance^2^ is furthering the involvement of young people in setting priorities for research in its association with the British Academy of Childhood Disability.

Children with life limiting conditions and their families have complex needs that require a range of skills and services in order for their needs to be met. Support is often required from a number of organisations within health care, social care, education and the voluntary sector. Most care, however, is still provided at home by parents (Carter and Coad, 2009; Department of Health, 2008; Hannan and Gibson, 2005; McIntosh and Runciman, 2008; Olsen and Maslin-Prothero, 2001). Due to the complex needs of these children and young people, it is essential that their views, along with those of their families, are embedded into service design and provision to ensure that services are relevant and appropriate. Review of services needs to be ongoing to ensure that families are receiving the care and support that they need (Craft and Killen, 2007).

The consultation presented in this study was based upon the former government’s strategy for children’s palliative care: ‘*Better Care: Better Lives*’ (Department of Health, 2008). This strategy called for the development of strong commissioning networks and a better understanding of local population needs in order for local commissioners to be able to develop more responsive and more sustainable services.

The West Midlands region provided the focus for this consultation and for the proposed Big Study. This region is large (equal to approximately one-sixth of England) and has strong children’s palliative care networks, with a variety of types of services and diverse ethnic communities. In collaboration with academic partners, ACT was successful in applying to the Big Lottery Research Programme for a Development Grant to enable preparation of a full bid for evaluation of services. The work proposed for the Development Grant included consultations with children, young people, parents and service providers.

## Aims

The aims of the consultation were to:

1. identify the important issues affecting families and providers of services in the area;
2. draw upon these issues in developing the bid for The Big Study;
3. identify organisations and individuals who might be interested in collaborating in The Big Study and those who might join Research Advisory Groups.

## Methods

The consultation for this study was based upon Oliver et al.’s (2008) definition of user involvement, terms being ‘consultation’ (seeking the views of users); ‘collaboration’ (more active and ongoing partnership) and ‘user-controlled research’ (user leading the research). INVOLVE (2012) has similar categories.

It was intended for the consultations to engage widely with both users and providers of services for children with palliative care needs and their families and for the consultations to be accessible to and appropriate for those with particular needs and vulnerabilities. Multiple methods of engagement were undertaken, including focus groups, individual interviews (either face to face or by telephone) and participatory activities for parents and children (Carter et al., 2002; Coad et al., 2009; Rollins, 2005; Save the Children, 2001). As much as possible, the settings for the consultations were made interesting and comfortable. (As seen later, more preparation in one of the settings was required than we had anticipated.) The level of PPI at this stage was predominantly ‘consultation’ (asking for views) (Oliver et al., 2008); first, to use findings from the consultation to develop the main research bid and second, to develop collaborative relationships with the children, young people, families and service providers to enable us to work in partnership in The Big Study.

### Ethics

The National Research Ethics Service (NRES) and Chair of the local Research Ethics Review Committee agreed that the proposed activities should be considered to be consultation and good practice prior to the development of the research proposal, rather than research itself, reflecting the INVOLVE and NRES position on PPI in research (INVOLVE, 2012). Consequently, National Health Service ethics approval was not needed for this preliminary work. However, the proposed methods for the consultation were approved by the Faculty Ethics Committee at the lead author’s university. Additionally, all researchers involved in this consultation had a recent enhanced Criminal Records Bureau clearance.

Prior to commencing consultations, participants were invited to sign a consent form to permit discussions to be recorded. Participants were assured that anything they said would be anonymised in any reports or other documents. Participants who preferred not to be recorded were given the option to talk individually to a researcher, with only written notes being taken. Participants were offered travel expenses.

As much was done as possible to ensure that the consultations with service users were fair and equitable. This included attempting to engage with people from ethnic minority communities, with those who spoke little or no English and with children with disabilities. Many children with life limiting conditions have learning and/or communication difficulties, and it was considered important not to exclude these children from the consultations. Members of the project team have considerable experience of engaging children with disabilities in consultations about issues that are important to them and using conversation and arts-based techniques to elicit their views (Brown and Warr, 2007; Coad et al., 2009).

It was important for local stakeholders (commissioners, service providers and families) to be kept informed about the consultation project and the anticipated Big Study. It was envisaged that professionals might have some uncertainty and anxiety about the research. It was our aim to set their minds at rest so they would be more prepared to be involved and help us to make contact with families.

### Design of consultations

Consultation and involvement of users early in the preparation and development of a research proposal provides a key opportunity for informing and influencing study aims, methodology and outcomes and, therefore, the relevance and impact of the research (Staniszewska et al., 2007). Three different types of consultation were held: (1) individual and/or group interviews with parents and service providers; (2) group interviews with children at school and (3) a focus group with service professionals. Participants were asked to address the following questions:

1. What questions would you like us to ask in the research?
2. What is important to you … in the services that families receive?
3. What would you like to change in services if you were able to?

In addition, children could also help to ‘design a study logo’ and ‘give a name to the study’.

#### Individual and/or group interviews with parents and service providers

Letters of invitation and information sheets were sent to parents known to ACT living in the West Midlands area. In addition, service providers were informed of the event and invited to attend and were also asked to make families known to their services aware of the consultation.

##### Open day

**Figure 1.**
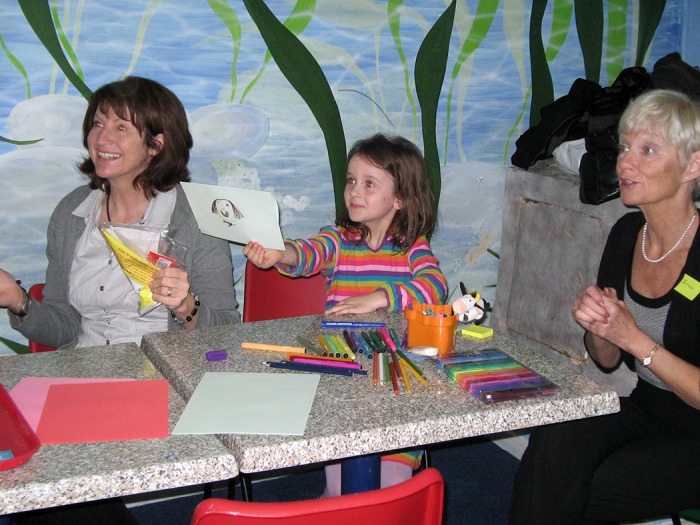
Part of research team ready for action at the aquarium (all pictured with permission).

An open day was held at a Sea-Life aquarium in Birmingham, West Midlands, England. The event was open to children, young people and their families and also for professionals who wished to attend. Activities were aimed at engaging participants in either group or individual discussions with researchers about services in the area (Figure 1). Refreshments were provided and participants were able to come and go throughout the day at their leisure.

##### Individual telephone interviews

Individual telephone interviews were also offered to families and service providers if they were either unable to attend the group activities or they preferred to talk alone with only the researcher listening.

#### Consultation with children at school

In addition to the event at the aquarium, an event was arranged at a local school for children with special needs, in which children and young people could take part in group activities with their peers – a situation in which they might have more confidence in relaying their views. Parents and young people at the school were invited by the head teacher, on behalf of the research team, to engage through conversation and art-based activities. Whilst most of the children and young people did have a life limiting condition, the head teacher did not feel it appropriate to only invite those with life limiting conditions so their confidentiality could be maintained. The young people were divided into two groups, with a researcher in each to facilitate activities. A third researcher moved between the tables, taking notes and providing individual help to young people who had more physical difficulty in participating. The children and young people discussed the questions listed on the previous page. The products from their discussions were notes on ‘post-it notes’ and postcards that were later grouped under inductively formed thematic categories by the researchers.

#### Focus group for service professionals

Professionals from the West Midlands Paediatric Palliative Care Network were invited via email and word of mouth to take part in a focus group held at the headquarters of one of the children’s hospices, which often hosted Network meetings. The majority of the service providers were represented on this Network. The focus group method was chosen as a means of both informing professionals of the proposed research bid and gaining their interest and participation. The aim of the focus group was to seek the views of the health-care professionals regarding which issues were relevant to the local area and regarding what they deemed to be questions of importance that the research might explore. Additionally, the group was asked for their advice on methods to engage service users and disseminate findings. Members of the group were invited to be part of a Professional Advisory Group supporting the development and conduct of The Big Study.

### Data analysis

All participants gave permission for interviews and groups to be digitally recorded. Recordings were not transcribed but notes made on the content. These notes, together with the data and notes made at the time of the activities, were analysed thematically with a view to drawing from them information that would guide the goals and design of The Big Study. The researchers compared analyses and discussed any areas in which there were differences in understanding so that consensus was achieved in deciding upon the goals and design of The Big Study.

## Findings

Overall, participants did not articulate research questions but focused on discussing, reflecting and recounting their experiences.

### Findings from the open day interviews

We do not know how many families were ultimately invited or otherwise heard about the open day. Bad weather and queues at the main entrance could have deterred many. Ultimately, only one family and one professional attended the open day. Although the affected child was not able to participate due to profound disability, both parents, who were from a minority ethnic background, and the child’s employed carer were interviewed, sometimes as a group and sometimes separately. A toddler sibling played alongside. Another parent who could not attend the open day was interviewed over the phone.

Although only two families participated, the data gathered from the parents’ interviews were insightful and valuable. Three main themes arose from the interviews. First, the parents felt that they had had to *fight for and justify their children’s and their own needs*. Second, the parents expressed *fear of loss of services* resulting from changes in their own circumstance, changes in local policies or reduction in service funding. Finally, it was felt that *decisions seemed to be made beyond and outside of their understanding* *and participation*, encapsulated by a mother’s statement ‘*I don’t know why it happens like this?*’ Similar experiences were described in a previous study (Hunt et al., 2003).

A recent Department of Health White Paper (Department of Health, 2010) suggests that patients will get more choice and control. This should be underpinned by an information revolution, enabling services to be more responsive to patients and to be designed around them, the principle being ‘no decisions about me without me’. This did not appear to have been the experience of the families who were consulted in either this study or, for the most part, in the previous study (Hunt et al., 2003).

### Findings from the consultation with children in school

Six boys and one girl (aged from 13 to 18 years) took part in the school activities. All but one of the young people had physical disabilities, including five who were wheelchair users. Discussions about issues that were important to the young people ensued, and in analysing the data, three key themes arose: ‘*quality of environment*’, ‘*quality of services*’ and ‘*quality of life*’. These three themes will now be discussed in more detail.

#### Theme 1: Quality of environment

As also reported by Coyne and Kirwan (2012) and Lambert et al. (2013), children and young people demonstrated an interest in the quality of hospital environments. In their discussions, young people compared two local hospitals of which most of them had experience, with one being favoured over the other because it appeared to be (and in fact had been) designed with children and young people in mind (Coad and Coad, 2008). ‘The building is modern – it is clean; well run’. Staff members at this hospital were also praised – ‘I like the doctors at the [place of hospital]. The nurses are friendly and I like the adolescent ward. It is nice’.

The children and young people also spoke of their school environment, arguing for keeping and refurbishing their current school rather than, as was planned, it being replaced in a different area of town. ‘My two problems with the school are it needs decorating in the rooms and the school meals look like they are over cooked – not the cook’s fault’. Another says ‘I would like the school decorated and have nice new flooring’. ‘I would wish for our school not being knocked down’.

#### Theme 2: Quality of services

Children and young people were appreciative of some services, for example, the school services and careers service, saying, for instance – ‘The teachers are world class’. ‘My school transport is always on time’. ‘The Connexions Adviser, [name], she is helping us with our future and she decides which college we would like to go to’. However, they also identified areas with which they were less content, for example, the council transport service, – saying ‘My main problem with the Council is their transport service. My driver and escort don’t talk to anyone. It looks like it was cobbled together at the last minute’.

The local accident and emergency (A&E) services were criticised, especially for long waiting times. ‘The waiting isn’t very good (at hospital) … you probably wait for 4 hours in A&E. I wasn’t very happy about that …’. Additionally, the children and young people experienced repeated cancellations of admissions for surgery, one young person saying ‘It (hospital) keeps cancelling (my operation) – 4 times. Not enough beds at [place of hospital]’. Children and young people wrote of the lack of available carers to support their parents, one saying ‘Mum and dad do all the care. No carers to help. Mum has a bad back. Dad lifts’.

During the school event, a discussion ensued about wheelchairs, with enthusiasm expressed for one participant’s new electric wheelchair. However, the participants also alluded to a lack of foresight and anticipation by some providers in relation to wheelchairs being outgrown. It is a frequent criticism that providers can be slow to respond to predictable changes such as the growth in height of a child or young person (Hunt et al., 2003).

#### Theme 3: Quality of life

The theme of ‘quality of life’ appeared to be centred on living as ‘normal’ and independent a life as possible, with young people saying, for instance, ‘My dream is to walk and run again’; ‘I would like to walk. My family would like that’; and ‘I would love to be independent and to go out by myself’. Additionally, being able to spend time with school friends during school holidays and spending time with family during days out seemed particularly important.

However, quality of life could be compromised when children and young people felt that they were being picked on or stared at. Children and young people appear particularly vulnerable to bullying or unwanted attention, for example, ‘People stare at me’; ‘People have picked on me in the past’; ‘People ask me why I can’t walk now. I say “leave me alone”’ and ‘I would wish for [.] all bullying stopped everywhere’.

A name for The Big Study ‘*About UZ and U2*’ (translated as ‘About Us and You too’) suggested by one of the young people (and to become the heading for the children’s and young people’s involvement in The Big Study), perhaps sums up the immense importance to the young people of the interactions between them and their physical and social worlds emphasising that policy needs to promote children’s and young people’s well-being both in the short-term and long-term (Carter, 2012; The Children’s Society, 2012).

### Findings from focus group for service professionals

Ten health-care professionals participated in the focus group, including paediatricians, a children’s hospice representative and representatives from three community children’s nursing teams. Four key themes emerged from the focus group data analysis: *meeting the needs of children and their families*; *variation in needs and locations*; *collaborating to meet needs* and *networking to sustain services*. These themes will now be explored in more detail.

#### Theme 1: Meeting the needs of children and their families

It emerged that, whilst most members felt that they and their services were driven by a desire to meet the needs of children and families, in practice not all care was led by this need. It was noted, for instance, that as a child’s condition deteriorated, it became more difficult to meet their increasing needs. For instance, as children became sicker and care more complex, they tend to be offered less short-break opportunities by statutory services.

Participants also believed that children and young people requiring palliative care tend to be nowadays more dependent than in previous decades, for example, there now appears to be a larger number of children who require invasive technology such as tracheostomy and ventilation to maintain their lives. Caring for such children and meeting the needs of their families are both demanding and complex.

#### Theme 2: Needs and locations vary

It was apparent that tensions existed between the strongly felt desire of group members to provide services that meet the needs of families and, in some areas, their capacity to do so. For example, the capacity to provide short breaks for children or to care for children in their own home at the end of life can be limited. For several of the localities, it was felt that adequate end-of-life care is only possible through the good will of nurses who provide services outside of and additional to their usual working hours.

An additional difficulty acknowledged was the significant variation in needs between families and over time, creating a requirement for a variety and range of services. Needs were also deemed to vary according to locality, as a result of the substantial differences between the rural and urban nature of different localities across the West Midlands. However, despite the variation, there was perceived to be a strategic drive to develop similar policies for practice across different localities, facilitated by the Networks. There appeared some resistance to this from representatives of more outlying services.

#### Theme 3: Collaborating to meet needs

It was suggested that there are some needs that may be better met on a regional rather than a local basis, For example, the regional Children’s Hospice provides short breaks for children and is deemed to be important by participants in helping local services to meet the needs of children and families. It was recognised that without support from voluntary sector organisations, such as the Children’s Hospice, the statutory sector organisations would be severely compromised.

#### Theme 4: Networking to sustain services

The focus group was held at a time when the then Strategic Health Authority (SHA)^3^ had withdrawn clerical support and funding for the regional Paediatric Palliative Care Network. Networks such as the regional Paediatric Palliative Care Network were considered to be important facilitators for developing strategies to meet family needs, for sustaining services and for advocacy for voluntary sector services in their dialogue with local primary care trusts about commissioning.

It was noted that the professional group, similar to the parents and young people, did not readily suggest research questions but presented issues or problems in need of solutions.

#### Other issues for forthcoming research

An interview with an individual health-care professional yielded some further key issues to explore in the forthcoming research study. First, ‘*who leads?*’ was thought to be a key theme to explore. Second, *communication* was considered a key area, particularly in relation to the transition of young people from children’s to adult services. Finally, the *assessment of children’s and family’s needs* was deemed important to address, particularly as a recent ‘gap analysis’ conducted by the Network has demonstrated areas of excellent practice but also areas of concern such as the failure to establish the Common Assessment Framework (DfES, 2004).

## Discussion

An overarching theme from the three consultation activities was ‘quality’: service providers wanted to provide quality services and families wanted and needed to receive quality services. In addition to lack of resources, lack of information and consultation can also lead to the children’s and families’ needs not being met. ‘Individual assessment of need’ could be the link between the quality service that the professionals seek to provide and the quality of service and quality of lives that parents, children and young people seek to experience. Quality in this context appears in keeping with its description in the Department of Health (2009) document ‘Putting Patients at the Heart of Care’ – ‘*Quality means becoming truly responsive to what patients, local communities and staff want and putting them at the heart of what we do*’ (Department of Health, 2009: 8).

The consultation process provided the research team with an enhanced understanding of the geographical territory in which they might work as well as the population with whom they might work. In addition, there were two research questions raised by this consultation:

1. To what extent are services designed and positioned to meet the needs of the children and young people?
2. To what extent are children, young people and their families consulted about what they need and want?

In relation to Oliver et al's (2008) categories of service user involvement, the consultation activities described have been classed as ‘consultation’, because the research questions that emerged were constructed by researchers based upon the issues that arose from consultations with participants, rather than being constructed by the participants themselves (Lloyd et al., 1996). That is, the shaping of The Big Study tended to come from the analysis of the data rather than direct feedback from the participants due to challenges in translating areas of concern in the field in to research questions. Further research might examine this more fully. However, the development of research questions and priorities needs to become more inclusive and transparent in the future, reflecting, for instance, methods advocated by the James Lind Alliance Priority Setting Partnerships (2009).

It is often difficult to determine when consultation efforts have been sufficient (Dickert and Sugarman, 2005), and currently, consultations have only been undertaken with a small number of those who provide services and with an even smaller number of those who receive them. It is important to consult with a whole group, which in this case is not only those who receive services but also those who might for any reason be excluded from or currently not accessing them (Bewley and Glendinning, 1994; Lloyd et al., 1996). The Big Study (named thus in relation to the smaller consultation) would strive to reach ‘harder to reach’ and marginalised populations (Cavet and Sloper, 2004; Coe et al., 2008; Curtis et al., 2004) attempting to include those families who were not identified as service users. Finding ways of doing this, however, remain challenging. It was anticipated that a significant proportion of children with life limiting conditions would be from minority ethnic communities (Devereux et al., 2004). Greater emphasis and resources would be needed in The Big Study to include non-English-speaking parents so that their needs could be heard and recorded (Gatrad et al., 2003; Worth et al., 2009). Additionally, as much as possible would be done to include and enable children with communication impairments to participate in the study (Watson et al., 2007).

In The Big Study, now completed (Hunt et al., 2013), the research team continued to be guided by service users and providers throughout. Consultation continued through feedback – between the research team and three advisory groups (consisting of parents, children/young people and professionals). Members of the advisory groups were regarded as partners in the research and as important contributors to the study.

## Conclusion

A series of consultations were undertaken in preparation for the research proposal and bid for funding. These consultations proved extremely helpful in shaping the research questions and research design. It was clear that The Big Study needed to consider geographical and economic aspects of service provision, in addition to individual experiences for its results to be transferable to other regions. It was also important for researchers to gain an understanding of the formal and informal professional networks of service providers, in order for the study to provide theoretical insights that can inform provision both in the West Midlands and elsewhere. Overall, it was important to build closer links with family users so that their experience, contribution and collaboration would underpin The Big Study.

## Notes

1. ACT was a UK umbrella organisation for children’s palliative care, now part of Together for Short Lives.

2. http://www.lindalliance.org/ChildhoodDisabilityPSP.asp.

3. In April 2013, Strategic Health Authorities were replaced by clinical commissioning groups and the National Health Service Trust Development Authority.
